# T167. ABERRANT MYELINATION OF THE CINGULUM BUNDLE IN PATIENTS WITH SCHIZOPHRENIA: A 7T MTI/DTI STUDY

**DOI:** 10.1093/schbul/sby016.443

**Published:** 2018-04-01

**Authors:** Lena Palaniyappan, Ali Radaideh, Olivier Mougin, Penny Gowland, Peter Liddle

**Affiliations:** 1 University of Western Ontario; 2 The Hashemite University; 3 University of Nottingham

## Abstract

**Background:**

The structural integrity of the anterior cingulum has been repeatedly observed to be abnormal in patients with schizophrenia. Reduced glutathione levels, indicating oxidative stress, is associated with reduced structural integrity of cingulum bundle in patients with schizophrenia. Variations in neuregulin-1, a well-established candidate marker for schizophrenia, results in oligodendrocyte dysfunction and defective myelination, and is shown to affect the structural integrity of the anterior cingulum in patients with schizophrenia. While the evidence to date has been obtained using diffusion tensor imaging, abnormal tract-specific changes in myelin content can be more directly inferred by combining multiple modalities of WM imaging such as diffusion tensor (DTI) and magnetization transfer imaging (MTI) in parallel.

**Methods:**

We used ultra-high resolution (7 Tesla) MTI in 17 patients with schizophrenia and 20 controls, to evaluate the macromolecular (predominantly myelin) content of the brain. Immediately after the 7T scanning, we also obtained a 3T diffusion tensor image (DTI) and undertook probabilistic tractography using FSL software (AutoPtx, ProbTrackX) to delineate anterior cingulum bilaterally. Unpaired t tests were used for group comparisons along with estimates of Cohen’s d or Hedge’s g for effect sizes.

**Results:**

Patients had a significant reduction in magnetization transfer ratio (MTR) in right (Cohen’s d=0.91, p=0.007) but not left (d=0.03, p=0.92) cingulum bundle. There was also a trend level reduction in fractional anisotropy of right (d=0.60, p=0.07) but not left (d=0.47, p=0.17) cingulum bundle. We did not find any significant relationship between the 3 major symptom dimensions of schizophrenia (Reality Distortion, Disorganization, Psychomotor Poverty) and Cingulum MTR. Patients with Schneiderian delusions (n=5) showed a significantly reduced MTR of left cingulum compared to patients (n=12) with no Schneiderian delusions (Hedges’ g=1.36, p=0.02).

**Discussion:**

Our findings suggest that MTR changes in anterior cingulum, resulting from either dysmyelination or neuroinflammation, is present in clinically stable patients with schizophrenia despite their medicated status. We lacked sufficient power to detect association between MTR changes of cingulum and symptom dimensions. Nevertheless, our results suggest that MTR changes are of higher magnitude than changes in fractional anisotropy, indicating the sensitivity of measuring myelination as a biological marker of white matter aberrations in schizophrenia.

